# A qualitative literature review exploring the role of the inguinal ligament in the context of inguinal disruption management

**DOI:** 10.1007/s00276-018-2170-6

**Published:** 2018-12-20

**Authors:** Andrew David Clelland, Ourania Varsou

**Affiliations:** 1 Edinburgh Medical School, University of Edinburgh, Edinburgh Bioquarter, 49 Little France Crescent, Edinburgh, Scotland EH16 4SB UK; 2 School of Medicine, University of St Andrews, North Haugh, St Andrews, Scotland KY16 9TF UK; 3 Anatomy Facility, School of Life Sciences, University of Glasgow, Glasgow, Scotland UK

**Keywords:** Inguinal ligament, Tenotomy, Inguinal disruption, Groin pain, Sportsman’s hernia, Denervation

## Abstract

**Purpose:**

Groin injury, sportsman’s groin and inguinal disruption (ID) refer to a diffuse chronic groin pain syndrome that has significant impact on athletes and is often unresponsive to conservative management. The ID aetiology is poorly understood but may involve weakness of the inguinal ligament attachments or the posterior inguinal canal wall or the tendons of adductor longus and rectus abdominis. We discuss the literature in which the inguinal ligament was directly targeted for ID management in athletic populations. Secondarily, we discuss the anatomical reclassification of the inguinal ligament to a tendon based on the above information.

**Methods:**

This was a qualitative review of the published literature, in English, from January 2007 to February 2017.

**Results:**

Five research papers, including 264 patients, were appraised. In patients with ID, tears were identified in the inguinal ligament, and to relieve pain, the surgical treatment of the ligament by tenotomy was shown to be beneficial. Techniques such as radiofrequency denervation involving the inguinal ligament and ilioinguinal nerve were also shown to relieve symptoms in athletes.

**Conclusions:**

This qualitative review has specifically focused on the literature directly targeting the inguinal ligament in ID which is a relatively unexplored management approach. When treated as a tendon, the inguinal ligament appears to be an appropriate ID therapeutic target. Integrated studies and randomised clinical trials will promote a better understanding of the role of the inguinal ligament and its tendinous properties in ID and provide a foundation for evidence-based management of chronic groin pain in athletes.

## Introduction

The inguinal ligament was first described by Fallopius in 1562 with its structure being further delineated by François Poupart in 1705 [15]. Cunningham discussed its ‘tendinous’ anatomy while spanning from the anterior superior iliac spine (ASIS) to the pubic tubercle (PT) with its upper surface being grooved due to the inrolling of the external oblique aponeurosis [5]. Lunn describes the medial half of the inguinal ligament as being more mobile due to the sole contribution of external oblique aponeurosis to this part [14]. Doyle showed that no part of this structure had any ligamentous thickening [6]. This evidence provides further support for therapeutically treating the inguinal ligament as a tendon.

Groin injuries account for 2–5% of all sport-related injuries [22], with an incidence of 5–18% among professional tennis and football players [32]. Injury patterns vary by sex with groin injuries being more prevalent amongst men [30, 31]. This is most likely multifactorial partly explained by the embryonic descent of the testes [8], higher torque experienced by the male musculature during certain sports, or differences in circulating oestrogen levels affecting connective tissues [18]. Inguinal disruption (ID) describes a syndrome occurring in amateur and professional athletes partaking in vigorous high intensity exercise that involves frequent kicking and twisting movements whilst running (i.e. football and hockey) [16]. Patients experience relapsing–recurring groin pain [23], which may be acute or chronic, and is clinically diagnosed following exclusion of all other obvious pathology [28]. Other terms describe the same syndrome, such as athletic pubalgia, sportsman’s groin and sportsman’s hernia, although a ‘true’ hernia is rarely present.

Inguinal hernias may present similar to ID, with pain radiating towards the groin which is worsened by raised intraabdominal pressure. Typically, indirect hernias occur in children under one and direct occur in adults over 45 years of age [11], cohorts which differ substantially from the young athletic populations affected by ID [19]. In practice, true inguinal hernias may be differentiated from ID through clinical history as well as the presence of a palpable mass on examination, which does not exist in ID. A 2014 consensus panel of multidisciplinary experts concluded that when presenting with symptoms suggestive of ID, patients should undergo magnetic resonance imaging (MRI) to exclude other causes of groin pain [28]. However, this investigation carries limited scope for positive diagnosis. Ultrasound may also help with establishing differential diagnosis especially occult inguinal hernias [33]. The panel proposed a management algorithm whereby those patients with pain suggestive of ID undergo a 2-month period of physiotherapy, prehabilitation, rest and analgesia, with either open or laparoscopic repair considered beyond this time period. However, conservative management is often not beneficial and if ID is left untreated, it causes significant physical limitation with a detrimental effect on an athlete’s ability to train and compete especially at a professional capacity.

The primary aim of this review was to discuss the published literature in which the inguinal ligament has been directly targeted as a tendon for therapeutic purposes in the management of ID amongst athletic populations. A secondary aim was to discuss the anatomical reclassification of the inguinal ligament to a tendon based on the above information and drawing from structural and functional evidence supporting this argument. The hypothesis was that direct inguinal ligament tenotomy [13, 19] or radiofrequency denervation (RFD) [4, 20] would be clinically beneficial in the context of ID management in athletes and that the published literature would support the reclassification of the inguinal ligament to a tendon.

## Methods

### Search strategy

**Fig. 1:**
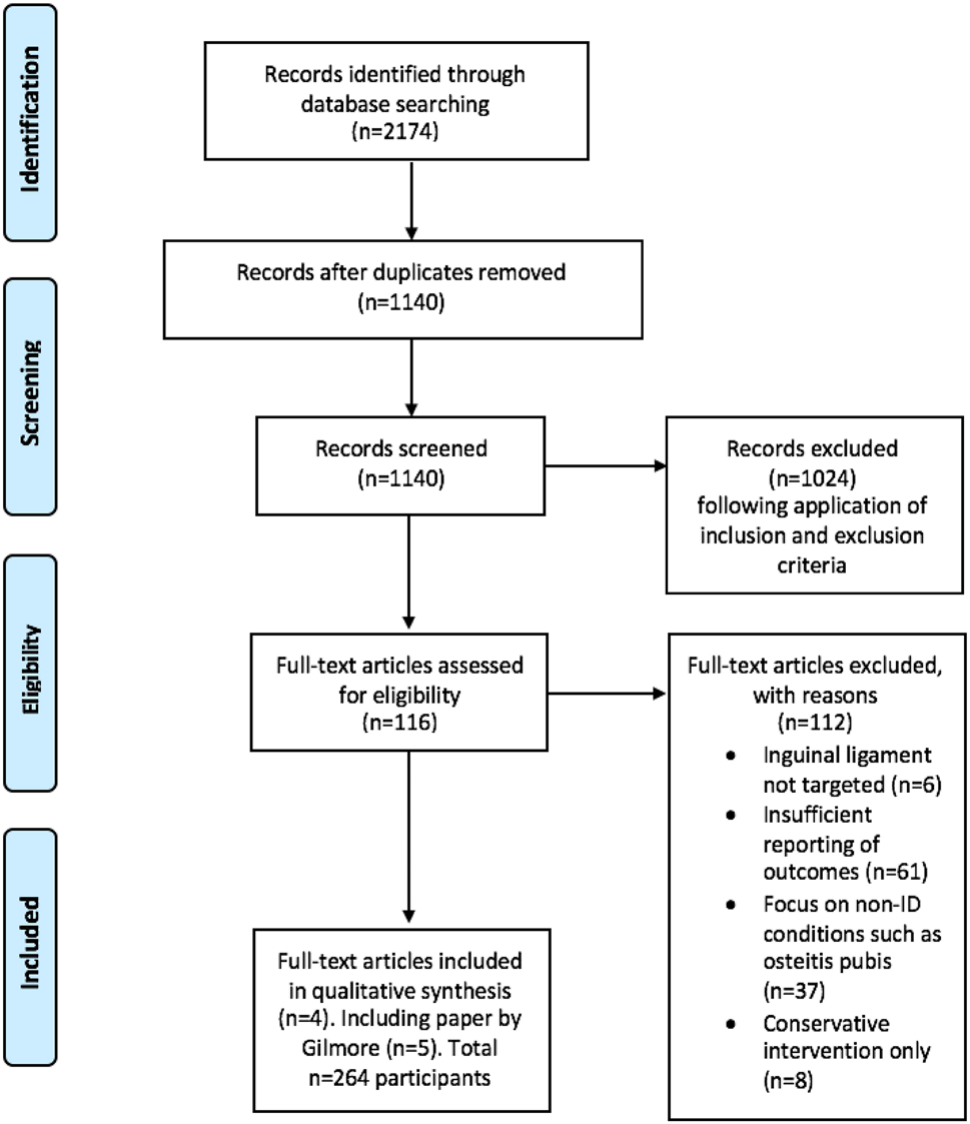
PRISMA (http://prisma-statement.org/) flow chart summarising the literature search strategies

Ovid Medline, PubMed, Embase, Cochrane Library (central register of controlled trials), SCOPUS (1960 onwards) and Web of Science (core collection) were searched for full-text primary research papers published in the English language from January 2007 to February 2017 (Table 1; Fig. 1). The following keywords were used: ‘sportsman groin’, ‘sportsman hernia’, ‘athlet* pubalgia’, ‘athlet* groin pain’, ‘symphysis syndrome’, ‘hockey groin’, ‘Gilmore* groin’, ‘adductor related groin pain’, ‘inguinal disruption’, ‘inguinal AND tendon’ and ‘inguinal AND tenotomy’.

**Table 1:** Inclusion and exclusion criteria

Criteria	Inclusion	Exclusion
Study population	> 50% athletic population with diagnosis of groin pain treated with surgical or radiological intervention	< 50% athletic population
Nature of management	Interventional (i.e. surgery or interventional radiology)	Non-interventional/conservative (i.e. pharmacological or physiotherapy only)
*n*	> 10	< 10
Study design EBM level (Ackley et al. [1])	Level II–IV evidence (i.e. RCT, cohort studies, case–control studies)	Level I and Level V + evidence (i.e. systematic reviews, meta-analyses, cross sectional studies, case reports)
Time period	January 2007–February 2017	Studies prior to 2007^a^
Outcome variables	At least one quantitative outcome measure (e.g. return to play rate, return to play time, treatment success, pain severity score, functional limitation score or patient satisfaction)	No quantifiable outcomes reported
Language	English only	Non-English language
Methodological quality	Adequate reporting of patient demographics, adequate description of intervention	Insufficient reporting of patient assessment or enrolment processes
Specific-related pathologies	Adductor tendinopathy Adductor-related groin pain	Osteitis pubis Meralgia paraesthetica Femoroacetabular impingement Anatomical variation studies
Publication status	Published (including ePub ahead of print) studies	Unpublished studies

^a^An exception was made for Gilmore which was the first paper in which the inguinal ligament was therapeutically targeted

### Study eligibility

Papers from database searches were collated and duplicates removed using Mendeley Desktop (Fig. 1). Titles and abstracts of 1140 records were screened and those which did not meet the inclusion criteria for study population demographics (< 50% athletes), number of participants (< 10) or management (conservative) were excluded. If it was unclear from the title and abstract whether studies were suitable, the full texts were reviewed. Following this step, the main text of 116 papers was assessed for eligibility against the full list of inclusion and exclusion criteria (Table 1). One reviewer performed the literature search at two separate time points (February 2017 and September 2017) and extracted the data (ADC).

Within the bounds of this review, four studies were identified as directly targeting the inguinal ligament in ID management for athletic populations. An important study conducted by Gilmore [8] fell out-with our inclusion criteria, due to its earlier date of publication, but it has been included in the review as it was the first paper to target the inguinal ligament in the context of ID and specialists in the field quote it extensively. A National Institute for Health and Care Excellence (NICE) algorithm for classifying study design was applied to each paper by one author (ADC). Each study was then assessed using appropriate Critical Appraisal Skills Programme (CASP) checklists, after which levels of evidence were designated according to Ackley et al. [1]. Due to the wide heterogeneity in outcome reporting amongst the identified studies, it was impossible to conduct a statistical analysis or a meta-analysis and for this reason the published literature has been appraised qualitatively.

## Results

In total, five primary research studies including 264 patients (89% male) directly targeted the inguinal ligament for ID management in athletes (Tables 2, 3). The first is by Gilmore that involved surgical repair of the conjoint tendon with plication to the inguinal ligament [8], followed by two studies involving laparoscopic transabdominal preperitoneal (TAPP) repair with inguinal ligament tenotomy [13, 19], and finally two studies targeting the inguinal ligament through RFD [4, 20]. Inguinal ligament tenotomy was performed on 121 patients (37/121, 31% professional athletes; 112/121, 93% male), and RFD involving the inguinal ligament and ilioinguinal nerve was performed on 60 patients (proportion of professionals could not be determined from available data; 60/60, 100% male). Therefore, the inguinal ligament was therapeutically targeted in 181 patients with ID, of whom 172 (95%) were male.

**Table 2:** Study design, participant demographics, intervention and outcome measures

Author year	Study design, EBM level (Ackley et al. [1])	Participant demographics	Intervention	Outcomes
Gilmore 1991 [8]	Retrospective cohort study Level IV	Study population: 65 professional association footballers (65/65, 100% professional athletes) Age: not stated Gender: 65/65 (100%) male Clinical presentation: mean duration of pain 22 weeks; all participants had chronic pain in the inguinal region; no visible or palpable signs in any patient	Repair and plicate conjoint tendon to inguinal ligament	Pain: not reported Return to play: average return to play time 6 weeks. 63/65 (97%) at 10 weeks; 65/65 (100%) at 2 years
Lloyd et al. 2008 [13]	Retrospective cohort study (with prospective questionnaire) Level IV	Study population: 48 participants (37/48 (77%) were amateur athletes and 11/48 (23%) were manual labourers) Age: median age 38.5 years (min–max range 24–71 years) Gender: 42/48 (87.5%) male Clinical presentation: median duration of pain 18 months; participants with pain > 8 weeks and refractory to conservative management; all participants had localised tenderness over the inguinal ligament and superficial inguinal ring	Laparoscopic TAPP repair with inguinal ligament tenotomy	Pain: pain severity pre-operative 7 to post-operative 3 (*p* < 0.0001); pain frequency score pre-operative 3 to post-operative 1 (*p* = 0.0012) Return to play: of 28/48 (58.3%) who returned questionnaires, 26/28 (92%) returned to normal sporting activity, although time period was not stated; return to strenuous sport median 28 days
Mann et al. 2009 [19]	Prospective cohort study Level IV	Study population: 73 athletes (37/73, 51% professional athletes) Age: median age 30 years (min–max range 16–50 years) Gender: 70/73 (96%) male Clinical presentation: median symptom duration 6 months with no improvement following conservative management, parenteral corticosteroids or previous surgery; all participants had symptoms suggestive of inguinal ligament pathology	Laparoscopic TAPP repair with inguinal ligament tenotomy	Pain: pain severity from pre-op 7 to post-op 0 (*p* = 0.005); improvement in frequency of pain (*p* < 0.001). 97% reported improvement in symptoms, 73% free of symptoms Return to play: 73/73 (100%) for all sports return to competitive play at median 28 days’ post-op; 54/73 (74%) reported themselves match-fit by 28 days (31/37 (84%) professionals); 64/73 (88%) reported a return to full-fitness at follow-up
Comin et al. 2013 [4]	Randomised controlled trial Level II	Study population: 46 participants (20/46, 43% professional athletes) Age: mean age 43.2 years (min–max range 18–67 years) Gender: 46/46 (100%) male Clinical presentation: participants had chronic groin pain > 6-month duration with no obvious structural cause refractory to conservative management	RFD of both ilioinguinal nerve and inguinal ligament versus local anaesthetic and steroid injection	Pain: group 1 (*n* = 18) significant improvement in mean visual analogue scale with activity (VASa) at 6 months’ post-treatment (*p* < 0.001) and visual analogue scale at rest (VASr) score at 6 months’ post-treatment (*p* < 0.001); group 2 (*n* = 18) significant improvement in mean VASa at 1-week post-treatment (*p* < 0.001) with no significant difference from baseline at subsequent measurements; group 3 (*n* = 10) significant improvement in mean VASa scores (*p* = 0.007) at 6 months’ post-treatment and mean VASr scores at 6 months’ post-treatment (*p* = 0.017) Return to play: return to play time not measured with functional limitation used instead
Masala et al. 2017 [20]	Prospective cohort study Level IV	Study population: 32 high performance athletes (32/32, 100% professional athletes) Age: median age 26 years (IQ range 18.3–33.7 years) Gender: 13/32 (41%) were male Clinical presentation: mean duration of pain 7 months refractory to conservative management; pain around pubic symphysis region with tenderness over the superficial inguinal ring	Pulse dose RFD targeting obturator nerve, genital branches of genitofemoral nerve, ilioinguinal and iliohypogastric nerves	Pain: significant improvement from baseline at 1 month (*p* < 0.0001), 3 months (*p* < 0.005), 6 months (*p* < 0.0001), 9 months (*p* = 0.008) Return to play: no reference of return to full, competitive activity; 24/32 (75%) participants started training or physiotherapy within days of treatment

**Table 3:** Summary of main demographic and outcome measures

Author, year	*n*	Male (%)	Professional (%)	Return to play	Pain severity (VAS)
Gilmore 1991 [8]	65	100	100	97% at 10 weeks	Not reported
Lloyd et al. 2008 [13]	48	87.5	0 (77% amateur; 23% non-athletic population)	92% at 28 days	7–3 (*p* < 0.0001)
Mann et al. 2009 [19]	73	96	51	100% at 28 days	7–0 (*p* = 0.005)
Comin et al. 2013 [4]	46	100	43	Not reported	Group 1: significant improvements in VASa (*p* < 0.001) and VASr (*p* < 0.001) Group 3: significant improvements in VASa (*p* = 0.007) and VASr (*p* = 0.017)
Masala et al. 2017 [20]	32	41	100	Not reported	Significant improvements at 1 month (*p* < 0.0001), 3 months (*p* < 0.005), 6 months (*p* < 0.0001), 9 months (*p* = 0.008)

### Gilmore’s groin repair

Gilmore retrospectively assessed clinical information from 65 male professional association footballers (100% professional athletes). All patients had chronic pain in the inguinal region, without any visible or palpable signs. In all patients, the conjoint tendon was surgically repaired and then approximated to the inguinal ligament. This intervention was considered successful in 63 of 65 (97%) based on the outcomes of return to training within 3 weeks and return to competitive play within 10 weeks. The average return to play time was 6 weeks (Table 2) [8].

### Laparoscopic TAPP repair with inguinal ligament tenotomy

Lloyd et al. retrospectively assessed the case notes of 48 patients (42/48 male, 87.5%), of whom 37 (77%) were amateur athletes and 11 (23%) were manual labourers, who underwent laparoscopic TAPP repair with inguinal ligament tenotomy. All patients suffered tenderness over the inguinal ligament. There were significant improvements in pain severity and functional limitation and 92% of 28 participants, who completed prospective questionnaire, had a median return to play of 28 days’ post-operation (Table 2) [13].

Mann et al. assessed 73 athletes (70/73 male, 96%), 37 (51%) being professional athletes, who underwent laparoscopic TAPP repair with inguinal ligament tenotomy. All patients had symptoms suggestive of inguinal ligament pathology. 97% reported an improvement in symptoms, with 73% being free of symptoms. Median return to competitive sports was 28 days (Table 2) [19].

### Radiofrequency denervation

Comin et al. included 46 male participants with 20 (43%) being professional athletes, who were randomly assigned to two groups of 18. Group 1 underwent RFD, and group 2 were treated with local anaesthetic (bupivacaine) combined with corticosteroid (triamcinolone) injection. A further 10 participants who underwent previous unsuccessful surgery were assigned to group 3 that underwent RFD. In group 1, RFD significantly improved pain severity scores from baseline in rest and during activity up to 6 months. Local anaesthetic and corticosteroid resulted in improved pain scores at 1-week post-treatment, however, this effect was transient and subsequent measures did not differ significantly from baseline. The non-randomised group, who underwent RFD, also showed significant and consistent improvement in pain scores from baseline to 6 months’ post-treatment (Table 2) [4].

Masala et al. performed pulse dose RFD on 32 high performance professional athletes (100% professional athletes; 13/32 (41%) male) and found significant improvements in pain scores from baseline to 9 months’ post-treatment. However, one patient received no pain relief following two treatments, although this patient had no evidence of pathology on MRI. 24 of the 32 participants commenced physiotherapy within days of treatment, although the number of days was not specified (Table 2) [20].

## Discussion

A literature search, from January 2007 to February 2017, was conducted to allow for the identification of recent and clinically up-to-date data in the relatively new field of the inguinal ligament treated as a therapeutic target for the management of ID in athletic populations. Overall, five primary research studies including 264 patients were assessed in which inguinal ligament tenotomy was performed on 121 patients (31% professional athletes) and RFD was performed on 60 patients.

### Current ID management

Current management begins conservatively with physiotherapy to improve pelvic stability [25] that may be followed by non-steroidal anti-inflammatory agents or corticosteroid injection into the origin of adductor longus. The latter is considered an appropriate target as it has been pathologically implicated in groin injuries [9, 21, 26]. Interventional strategies, such as nerve blockade or surgery, may be considered after 2–3 months of unsuccessful conservative management. Novel treatments such as RFD may also be employed, although the exact mode of action is not yet fully understood [4].

Surgical interventions may be performed open or laparoscopically using a polypropylene mesh in either TAPP or totally extraperitoneal (TEP) repair. A non-randomised study has shown that minimally invasive techniques reduce return to play time when compared to open approaches [10]. The rationale for mesh placement is based on increased strain from several sources which converge on the PT. Theoretically, supporting this area should reduce strain and the long standing inflammatory process which had become pathological, thereby promoting recovery. Several operative adjuncts have been carried out by surgeons working in this area, such as adductor and inguinal ligament tenotomy. The rationale behind tenotomy of adductor–abdominal complex lies in a transmission of load to the pelvis, away from the lower limb. The subsequent reduction in muscle pull allows similar reduction of inflammation and subsequent healing of the enthesopathic tendon insertion [2], reducing pain and returning function. The above approaches highlight the variability in current practices when it comes to ID management.

### Pain presentation in reviewed studies

In the study by Gilmore, all 65 football players were described as having ‘Gilmore’s Groin’, experiencing chronic inguinal region pain which was made worse by actions such as sprinting, kicking, coughing and sneezing [8]. In Lloyd et al. and Mann et al., to confirm inguinal ligament pathology, patients must have had tenderness near the insertion of the inguinal ligament onto the PT or superficial inguinal ring without any evidence of a hernia. Although these stringent criteria may have excluded potential participants from the study due to varying pain presentations, the outcome measures from the studies by Lloyd et al. and Mann et al. were comparable to high level outcomes in existing literature for surgical interventions managing ID [24]. Pain severity scores improved slightly in the study by Mann et al. [19], possibly explained by access to higher quality physiotherapy programmes in the all-athletic population (51% of whom were professional), or by slight improvement in intra-operative technique over time. Comin et al. studied participants who suffered chronic groin pain of no clearly identifiable cause, evaluating whether RFD of the inguinal ligament and ilioinguinal nerve was superior to conservative therapy comprised of local anaesthetic bupivacaine and corticosteroid triamcinolone [4]. Those who underwent RFD in Masala et al. experienced pain around the pubic symphysis and tenderness over the superficial inguinal ring [20]. None of the 264 patients were reported to have a demonstrable lump upon cough impulse, which is a cardinal feature of inguinal hernias [11]. The studies included in this review support existing literature in that presentation of ID is highly variable and hence diagnosis is difficult [7]. As such we believe that any intervention—particularly surgical—must be tailored to each individual patient’s clinical presentation and hence suggested underlying pathology.

### Treatment approaches in reviewed studies

Gilmore managed patients by surgically repairing the inguinal ligament with plication to the conjoint tendon, with a focus on restoration of normal anatomy involving a six-layered surgical technique [8]. The author concludes that for some patients, conservative management will never be effective until the underlying dehiscence between the inguinal ligament and conjoint tendon is addressed [8]. Lloyd et al. and Mann et al. postulated that the inguinal ligament may be the underlying cause of ID due to its attachment to musculature of the anterolateral abdominal wall and as such considered inguinal ligament tenotomy an effective treatment of ID [13, 19]. In both studies the same surgeon performed laparoscopic TAPP procedure with inguinal ligament tenotomy by hook diathermy. The inguinal ligament was completely separated at its medial end from the PT, whilst the lacunar and pectineal ligaments were also divided if found to be thickened [13, 19]. The possibility remains that mesh repair and subsequent alleviation of strain on the abdominal wall muscles may be the primary reason for the success of these studies. In the study by Mann et al., 11 participants (15%) underwent operations previously for groin injury (six Gilmore procedures, six inguinal hernia repairs and two adductor tenotomies) to no effect [19]. This suggests that inguinal ligament tenotomy has most likely been the determining factor in symptom alleviation, warranting further investigation of the inguinal ligament and its targeting in therapeutic procedures. One major issue with this surgical approach is the potential for post-operative herniation, however, Lloyd et al. and Mann et al. postulate that this can be addressed with the application of a polypropylene mesh. Additionally, whilst one would expect inguinal tenotomy with mesh repair to potentially destabilise the inguinal region, as division of a musculoskeletal ligament would lead to such a speculation, these studies have reported improved outcome measures for athletes including return to play time and rate. This suggests that a reinforced inguinal ligament withstands high forces while transmitting these from the muscles to the attaching bones which are forces experienced by professional athletes. These features are of a tendon rather than of a ligament [12], questioning the ligamentous classification of the inguinal ligament. We suggest an alternative possible way to circumvent the risk of herniation by performing a partial inguinal ligament tenotomy, whereby only aberrant attachments to bone are released. As yet, there is no published data evaluating this surgical intervention.

For the patients with ID who did not undergo surgery, the results of the studies by Comin et al. and Masala et al. demonstrated that neuromodulation by RFD is effective in reducing pain. Masala et al. show that denervation around the groin area is effective in the short term [20], whilst Comin et al. provide evidence that the inguinal ligament is involved in the underlying pathology of ID [4]. Given that Comin et al. successfully treated what is described as a tendonitis of the inguinal ligament, we believe this provides additional evidence of the tendinous properties of the inguinal ligament.

### Suggested approach to managing unexplained athletic groin pain

As recommended in the 2014 multidisciplinary consensus, we believe that athletes with unexplained groin pain causing functional limitation should undergo an initial treatment period involving 2 months of tailored physiotherapy, prehabilitation, analgesia and rest, utilising a multidisciplinary approach [28]. Those whose symptoms extend beyond 2–3 months should be investigated by MRI with orthopaedic review to exclude relevant orthopaedic conditions. The scan may also demonstrate pathologies associated with ID such as tendinopathies of adductor longus and rectus abdominis as they insert upon the pubis [20]. Athletes who remain symptomatic should be seen by an experienced general surgeon for thorough clinical examination to elicit any tenderness over the inguinal ligament or its attachments in addition to the superficial and deep inguinal rings and exclude the possibility of herniation. Patients should then be considered for surgical intervention, with operative adjuncts taking into consideration each individual’s clinical signs and investigative findings.

### Inguinal ligament anatomy

The pubic bone is important in ID as the site of attachment of several structures which, if damaged, may precipitate a sport injury. From the PT, the inguinal ligament continues as the pre-pubic aponeurotic complex (P-PAC) which connects to the underlying fibrocartilaginous disk of the pubic symphysis. In addition, the pubic bone receives the tendons of adductor longus and rectus abdominis. It has also been suggested that ID is the result of a weakness or tear in the posterior wall of the inguinal canal [28]. Another possible mechanism is imbalance of force across the pubis due to the strong adductor muscles opposing the comparatively weak lower abdominal muscles, creating a shearing force across the hemipelvis [7]. This results in weakening of the posterior wall of the inguinal canal and enthesopathy—disorder of attachment—of the rectus tendon.

Gilmore describes a pathophysiological mechanism of ID comprising a torn external oblique aponeurosis causing a dilated superficial inguinal ring, in addition to dehiscence between the inguinal ligament and a torn conjoint tendon (sometimes called ‘Gilmore’s Groin’) [8]. It has also been suggested that an avulsion injury to the P-PAC could result in microavulsion of the inguinal ligament medially [17]. There is intra-operative photographic [13] and ultrasound data [4] that the inguinal ligament itself was torn medially in patients with ID.

### Tendons and ligaments

One might argue that the taxonomy—tendon versus ligament—of the inguinal ligament is purely semantic as they are broadly similar types of connective tissue. Macroscopically, these are classified according to their connections; either bone to bone for ligaments or skeletal muscle to bone for tendons. However, this rudimentary logic cannot be applied to the inguinal ligament, which connects the ASIS to PT whilst arising from the aponeurosis of the external oblique. Another consideration is that the purpose of a musculoskeletal ligament is to stabilise a joint between two bones. Although separate in the pre-adolescent, the ileum and pubis are fused as the singular hip bone in the mature adult. Resultantly, the inguinal ligament has no joint to stabilise per se.

Although ligaments and tendons comprised of the same basic components, the exact proportion and arrangement of each of these varies histologically according to the mechanical and functional needs of structures [27]. Amiel et al. noted significant histological differences between tendons and ligaments of ovine tissues. The authors reported that tendinous structures such as the patellar and Achilles tendons were hypocellular compared with the ligaments of the knee [3]. Amiel et al. also discussed that tendons contained a significantly higher total collagen concentration. Although both tendons and ligaments contain type I collagen, ligaments contain a higher proportion of type III collagen (10%) that is found in embryonic tissues than tendons (5%) [3]. This disproportionate amount of collagen may account for the functions of tendons including shock absorption and tension transfer from muscle to bone [12]. Historically, the literature has dictated that ligaments are for stability and must always be preserved (i.e. never divided surgically). As surgeons have now started performing tenotomy on the inguinal ligament, this raises questions over this structure’s anatomical classification and the implications for clinical practice.

### Strengths and limitations

Although there is literature relating to the treatment of ID in elite sportspeople, particularly utilising mesh repair procedures and adductor longus tenotomy [9, 21, 26], direct targeting of the inguinal ligament for therapeutic purposes is a relatively new and unexplored innovation and this review provides novel insights into this topic.

Reported differences in the discussed studies could be due to the intervention allocation itself rather than the actual intervention. As such, outcome data for interventions performed by Lloyd et al. and Mann et al. are only applicable to patients with symptoms and characteristics similar to those within these studies. This review has also highlighted widespread heterogeneity of outcome reporting. Currently, there is no consensus on the most appropriate outcome measures for athletic groin pain. Outcomes most commonly reported are on pain severity by visual analogue scale, return to play time and return to play rate. There is, however, an alternative patient-reported outcome questionnaire based on assessment of pain, symptoms and function which has been validated in young to middle-aged patients [29]. Our view is that there must be more consistent reporting based on these criteria to minimise data heterogeneity.

## Conclusions

This review has provided pooled evidence supporting the concept that targeting of the inguinal ligament, as a tendon, is beneficial in terms of reducing symptoms and improving return to play time in athletes experiencing ID. We support the 2014 multidisciplinary consensus, according to which athletes with clinical symptoms or investigative findings suggestive of ID should undergo a period of physiotherapy, prehabilitation, analgesia and rest during the diagnostic process with surgery considered if symptoms persist beyond 2–3 months. Surgical interventions should be tailored to each patient’s clinical presentation, investigative findings and hence likely underlying pathology. The development and validation of a reporting tool for patient outcomes will also allow for standardisation and undoubtedly aid the above process. Clinically integrated research studies are also needed to delineate the precise role of the inguinal ligament and its tendinous properties in ID. Future large scale randomised clinical trials should evaluate laparoscopic TAPP with and without inguinal ligament tenotomy to provide high quality evidence-based guidelines for the management of chronic groin pain in athletes.
